# Fibrin-Platelet Clots in Acute Ischemic Stroke. Predictors and Clinical Significance in a Mechanical Thrombectomy Series

**DOI:** 10.3389/fneur.2021.631343

**Published:** 2021-04-20

**Authors:** Francisco Hernández-Fernández, María E. Ramos-Araque, Rosa Barbella-Aponte, Juan David Molina-Nuevo, Jorge García-García, Oscar Ayo-Martin, María José Pedrosa-Jiménez, Lorena López-Martinez, Gemma Serrano-Heras, Enrique Julia-Molla, Tomás Segura

**Affiliations:** ^1^Department of Neurology, Hospital General Universitario de Albacete, Albacete, Spain; ^2^Department of Neurology, University Hospital of Salamanca, Biomedical Research Institute of Salamanca, Salamanca, Spain; ^3^Department of Neurology, University Hospital of Valladolid, Valladolid, Spain; ^4^Department of Surgical Pathology, Hospital General Universitario de Albacete, Albacete, Spain; ^5^Department of Radiology, Hospital General Universitario de Albacete, Albacete, Spain; ^6^Research Unit, Hospital General Universitario de Albacete, Albacete, Spain; ^7^Instituto de Investigación en Discapacidades Neurológicas (IDINE), Facultad de Medicina, Universidad de Castilla-La Mancha, Albacete, Spain

**Keywords:** stroke, thrombectomy, fibrin, thrombolysis (tPA), mortality

## Abstract

**Introduction:** The histological composition of the clot influences its mechanical properties, affects the efficacy of endovascular treatment (EVT), and could determine the clinical outcome of patients with acute ischemic stroke (AIS). Insights into clot composition may guide therapeutic decision-making prior to EVT and facilitate revascularization therapies.

**Material and Methods:** Consecutive patients with AIS recorded in a prospective single-center reperfusion registry from December 2015 to December 2019 and treated with EVT were included. Baseline, laboratory [including post-procedural C-reactive protein (CRP)], radiological, and angiographic variables were analyzed. We aimed to study the relationship between histological composition of the clot with basal neuroimaging, laboratory markers, and recanalization technique. The secondary outcome was to analyze the correlation between clot composition and functional outcome at 3 months assessed by the modified Rankin scale (mRS).

**Results:** From the study period, 360 AIS patients treated with EVT were included, of whom 189 (53%) fulfilled the inclusion criteria. One hundred (53%) cases of fibrin-predominant clot (FPC) were recorded. Full recanalization in FPC cases was achieved with higher probability when stent retrievers (SR) were selected as the first-line device (68.2%, *p* = 0.039). Patients with FPC had higher levels of CRP (*p* = 0.02), lower frequency of the hyperdense middle cerebral artery (HMCA) in baseline imaging (*p* = 0.039), and higher rates of mortality (*p* = 0.012). The multivariate analysis showed that the absence of HMCA (OR = 0.420; 95% CI 0.197–0.898; *p* = 0.025) and higher levels of CRP (OR = 1.01; 95% CI 1.003–1.019; *p* = 0.008) were predictors of FPC. Leukocytes and platelet counts were not associated with clot histology.

**Conclusions:** The absence of HMCA and higher levels of CRP were markers of FPC. In patients with FPC, complete recanalization was most likely to be achieved when a SR was selected as first line of treatment. Mortality was higher in patients within this histologic group.

## Introduction

Endovascular treatment (EVT) has become the standard of care in patients with acute ischemic stroke (AIS) and large-vessel occlusion (1). Complete recanalization and first-pass recanalization have been shown to be independent predictors of good functional outcome (2–4). However, these endpoints are currently achieved in <50% of patients, probably due to inadequate patient selection or factors related to the procedure (5).

One of the main reasons for the lack of efficacy of EVT is the scant knowledge of the mechanical properties of the clot (6–8) and its histological composition. Both factors are relevant to attainment of successful reperfusion (9) and could aid in the development of endovascular thrombectomy devices.

Based on the predominant cell type, clots are usually divided into red clots (RPC), white or fibrin-rich clots (FPC), and mixed clots (MC) (10). In addition, the distribution of the fibrin network (6) and platelet clumps can be highly variable (11). Therefore, some FPC are more resistant to revascularization than others due to their higher friction coefficient (7, 8, 12). On the other hand, RPC demonstrate a lower density and are more easily removed as a result. A better understanding of the histological composition of the clot could facilitate better clinical and angiographic outcomes.

We aimed to study the relationship between histological composition of the clot with basal neuroimaging, laboratory markers, and recanalization strategies including intravenous thrombolysis and clot retrieval devices. The secondary outcome was to analyze the correlation between clot composition and functional outcome at 3 months as assessed by the modified Rankin scale (mRS).

## Materials and Methods

### Study Design and Patient Selection

We performed a retrospective, observational, unicentric study based on a prospective registry of all consecutive AIS patients treated with reperfusion therapies in a tertiary stroke center, between 15 December 2014 and 15 December 2019. All patients included were clinically managed according to our institutional protocols, which are based on current international stroke guidelines.

The study was approved by the local Clinical Research Ethics Committee with reference number 2019/03/031. Written informed consent was obtained from all included patients or their relatives permitting entry of their information into our reperfusion registry and subsequent use of the data for scientific purposes, in accordance with the Spanish Personal Data Protection law.

**Figure 1:**
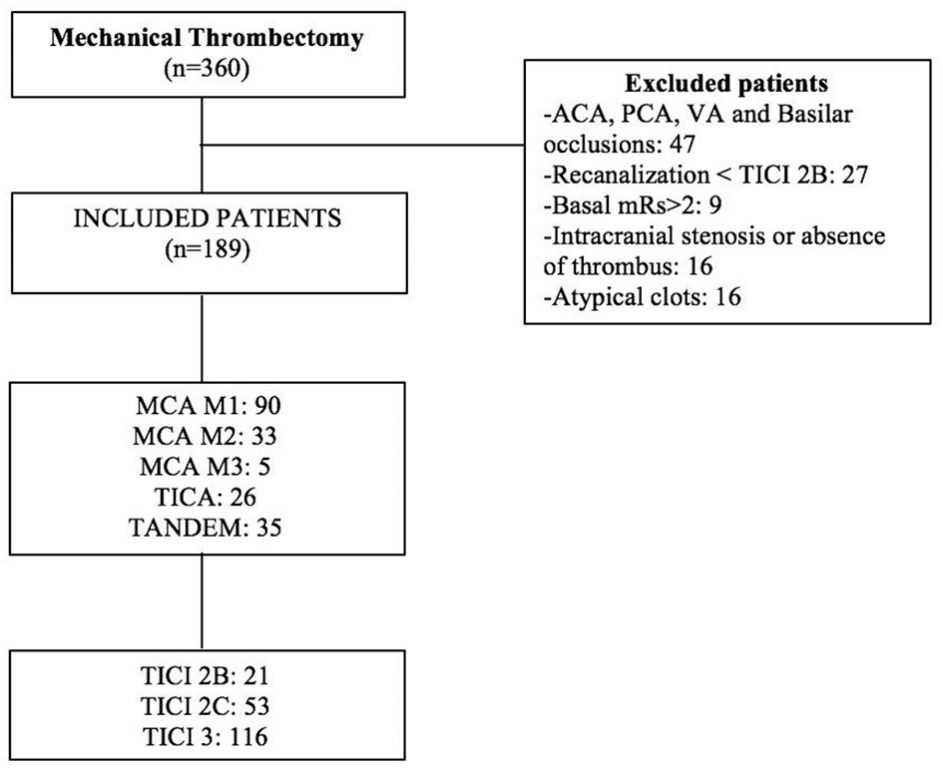
Flowchart of the patients included in the study.

Patients treated with EVT in our center were selected for this study if they fulfilled the following criteria: (1) substantial neurological deficit and ischemic stroke with demonstrated vessel occlusion; (2) time from symptom onset to groin puncture <24 h, including wake-up strokes and those of unknown onset; (3) no intracranial hemorrhage at baseline cranial tomography (CT); (4) no extensive early ischemic signs as defined by ASPECTS (Alberta Stroke Program Early CT Score) > 5; (5) in patients with time from symptom onset to EVT > 6 h, presence of target mismatch profile on CT perfusion; (6) occlusion in anterior circulation and absence of multiple-territory involvement; (7) effective recanalization (TICI ≥2B) and thrombus for histological analysis; and (8) absence of previous relevant disability as defined by the modified Rankin scale (mRs) (pre-stroke mRs score ≤2). If no contraindication existed, previous treatment with intravenous recombinant tissue-type plasminogen activator (IV r-tPA) was administered before EVT (Figure 1).

### Clinical Data and Baseline Variables

Our reperfusion registry includes data relating to the following baseline variables: age, sex, previous functional disability assessed by mRS, information about previous arterial hypertension, diabetes, dyslipidemia, coronary artery disease, atrial fibrillation, prior stroke, previous use of anticoagulants, or antiplatelet therapy. Administration of intravenous (IV) thrombolysis and etiology of the stroke based on the TOAST (trial of ORG 10172 in acute stroke treatment) classification (13) were recorded. Information regarding clinical stroke severity was assessed by the NIHSS (National Institute of Health Stroke Scale) on admission and during hospitalization. Urgent (blood count, biochemistry, and coagulation) and in-hospital laboratory tests were performed after EVT [biochemistry including thyroid hormones, lipid profile, and C-reactive protein (CRP)]. The white cell count was detailed, and the ratio of neutrophil and lymphocyte numbers was calculated.

Times from symptom onset to the CT, onset to groin puncture and procedural times (time from groin puncture to the end of the endovascular procedure), and type of device used (SR, aspiration, or combination) were registered. Number of passes and degree of recanalization by TICI (Thrombolysis in Cerebral Infarction) scale were obtained; TICI ≥ 2B was considered as effective recanalization. Prognosis at 3 months was evaluated by mRS considering good prognosis as mRS ≤ 2 and mortality mRS = 6.

### Neuroimaging Protocol

A basal cranial non-contrast CT was performed before EVT. Data on early ischemia was evaluated on basal CT by ASPECTS. Arterial occlusion was assessed on angio-CT scan and mismatch by CT perfusion.

All CT imaging was performed using a Philips Brilliance CT, 64-slice (Koninklijke Philips Electronics N.V., Amsterdam, Netherlands), and images were analyzed by a neuroradiologist.

At 24 h from the procedure or in the case of neurological deterioration, a control CT was performed to evaluate the presence of established infarct or hemorrhagic transformation. Symptomatic intracranial hemorrhage (PH-2) was defined according to Heidelberg criteria (14) as any intraparenchymal hemorrhage associated with an increase in the NIHSS scale score ≥ 4 points or death.

All baseline CTs were reviewed retrospectively on a blind basis by two independent neuroradiologists. The presence or absence of the hyperdense middle cerebral artery (HMCA) sign was classified by consensus based on visual inspection and according to the following criteria: (15) (1) spontaneous visibility of the MCA, (2) attenuation of the MCA greater than in the surrounding parenchyma, (3) disappearance in the bone window, (4) unilaterality, and (5) absence of subarachnoid bleeding. In case of disagreement, the case was discussed until a consensus was reached. Hounsfield units were also measured, and the ratio of healthy to pathological radiodensities was also calculated. The final value was considered as the arithmetic mean of the values obtained by each observer.

### Endovascular Procedures

The endovascular procedures were performed using a single-plane angiography, Innova GE model (General Electric Company, Schenectady, NY, US) or an Azurion 7 B20/15 biplane model (Koninklijke Philips Electronics N.V., Amsterdam, Netherlands) by one board-certified neurologist and a neuroradiologist. General anesthesia was used systematically.

Endovascular procedures were performed adhering to the following general methods:

1. Vascular access by puncture of the right femoral artery using the Seldinger technique. Placement of an 8F femoral introducer.
2. Selective catheterization using a Terumo 0.030″ guidewire (Terumo Corporation, Shibuya, Tokyo, Japan) and a Flowgate 8F balloon guide catheter (Stryker Corporation, Kalamazoo, MI, USA) and confirmed diagnosis of the affected territory. Placement of a guide catheter in the cervical segment of the ICA in anterior circulation stroke.
3. Use of stent retrievers (SR). Hyperselective catheterization of the occluded vessel and crossing of the clot with Traxcess 0.014″ microguide (MicroVention Inc, Tustin, CA, USA) and Rebar (Medtronic, Dublin, Ireland) or Trevo Pro (Stryker Corporation, Kalamazoo, MI, US) 0.021″ microcatheter. Placement of Solitaire FR (Medtronic, Dublin, Ireland), Trevo XP (Stryker Corporation, Kalamazoo, MI, USA), or Embotrap II (Johnson & Johnson, New Brunswick, NJ, USA) 4–6 mm SR in M1, M2, and intracranial ICA occlusions. In M2 occlusions immediately anterior to the opercular gyrus or M3 occlusions a Trevo XP or Catch mini (Balt, Montmorency, France), 3 mm SR was used. The device is removed after 3–5 min of balloon occlusion, and simultaneous manual aspiration was performed with a 60-cc syringe. Other devices used to a lesser extent were Revive SE (Johnson & Johnson, New Brunswick, NJ, USA) and Tigertriever (Rapid Medical, Yokneam, Israel).
4. Use of thrombus aspiration. Alternatively, in M1 occlusions, or after SR failure following two passes, an ACE64 or ACE68 (Penumbra Inc, Alameda, CA, USA) aspiration catheter is used, connected to an automatic pump for 3 min. Removal of the device and simultaneous manual aspiration with a 60-cc. syringe. In case the outcome was not satisfactory after two aspirations, combined use of SR and local aspiration is undertaken.After each pass, the device, intermediate catheter if withdrawn, and aspiration syringe were inspected for the presence of thrombus fragments. The device was gently washed with heparinized saline to remove thrombus fragments. Aspirated material was gently flushed with saline to identify any smaller fragments.
5. Control angiographic series in anteroposterior and lateral projections. Determination of the TICI scale.

### Histopathological Analysis

**Figure 2:**
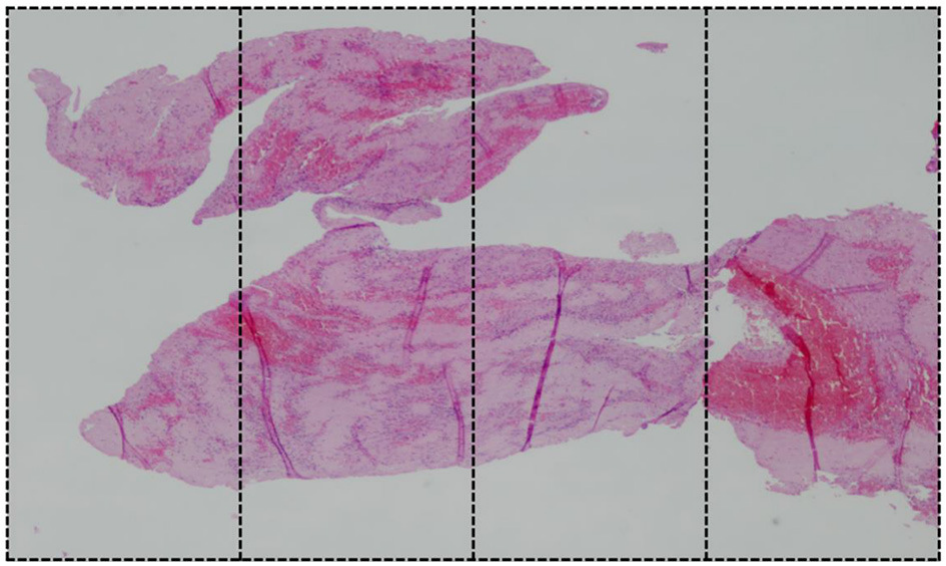
Mixed clot with predominant component of fibrin (80–85% FPC component). For histological assessment, the image is divided in four sections.

The clot was stored in 4% formaldehyde and sent to the Pathology Department, where it was embedded in paraffin, microtomed into 4-μ sections, and prepared using the established histological protocol, with hematoxylin–eosin, Gram, and Gomori's Trichromic staining. The histopathological study was performed by an expert neuropathologist, who analyzed the macro and microscopic characteristics of the clot, including red cell, white cell, and fibrin distribution. The semiquantitative percentage of these components was estimated, considering a clot either FPC or RPC when the percentage of these components was >60% (16–18). The rest of the clots, with no clear predominance of these components, were classified as MC. The general methods of this analysis are summarized in Figure 2. The clots were evaluated microscopically, and, whenever >3 mm in size, a subjective estimation was made of the red cell component and the fibrin and platelet component. The quantity of polymorphonuclear cells contained in the clot and their status were also evaluated. The use of gram, trichromic, and PAS staining allowed the presence or not of bacteria to be determined, as well as characteristics suggesting vascular recanalization.

### Primary and Secondary Outcomes

The main outcome was to study the relationship between histological composition of the clot with basal neuroimaging, laboratory markers, and recanalization strategies including intravenous thrombolysis and clot retrieval devices. The secondary outcome was to analyze the correlation between clot composition and functional outcome and mortality at 3 months assessed by the modified Rankin scale (mRS).

### Statistical Analysis

A descriptive study of the variables was performed using central and scattering tendency measures for quantitative variables and the exact calculation and percentage for qualitative variables. Normality of the sample was established using the Kolmogorov–Smirnov test. The comparison of the categorical variables between two or more subgroups was performed using the Pearson's chi-squared test or, as applicable, the Fisher exact test. The comparison between quantitative variables was performed with the Student's *t*-test. The comparison between medians was performed using the Wilcoxon signed-rank test. The independent effect of the clinical variables was calculated with multivariate binary logistic regression models, considering the presence of an FPC clot (bad outcome) as a dependent variable. The Hosmer–Lemeshow goodness-of-fit test was used to evaluate the global model fit. The variables included in the multivariate analysis, in addition to the time from onset of symptoms, were those with statistical significance *p* < 0.1 in the bivariate models. The odds ratio (OR) was calculated for each variable. A ROC (receiver operating characteristic) curve was performed to analyze the cutoff point with the best discriminatory value of the quantitative variables. A *p* < 0.05 was considered significant for all analyses.

All the results were analyzed with the statistical software IBM SPSS Statistics® Version 26 (SPSS, Chicago, IL, USA).

## Results

In the study period, 360 mechanical thrombectomies were performed (Figure 1). One hundred eighty-nine (52.5%) fulfilled our inclusion criteria. Reasons for exclusion were vertebrobasilar or multiple-territory occlusion (*n* = 47), absence of thrombus for histological analysis (*n* = 35), non-effective recanalization (*n* = 27), atypical clots: calcium, septic, or fat (*n* = 16), baseline mRS > 2 (*n* = 9), and incomplete clinical follow-up (*n* = 23).

The mean overall age of the cohort was 69.5 years (range 28–91); one hundred (53%) of the patients were male. According to the stroke etiology, 111 (59%) were cardioembolic, 42 (22%) indeterminate, 27 (14%) atherothrombotic, and 9 (5%) due to carotid dissection.

**Figure 3:**
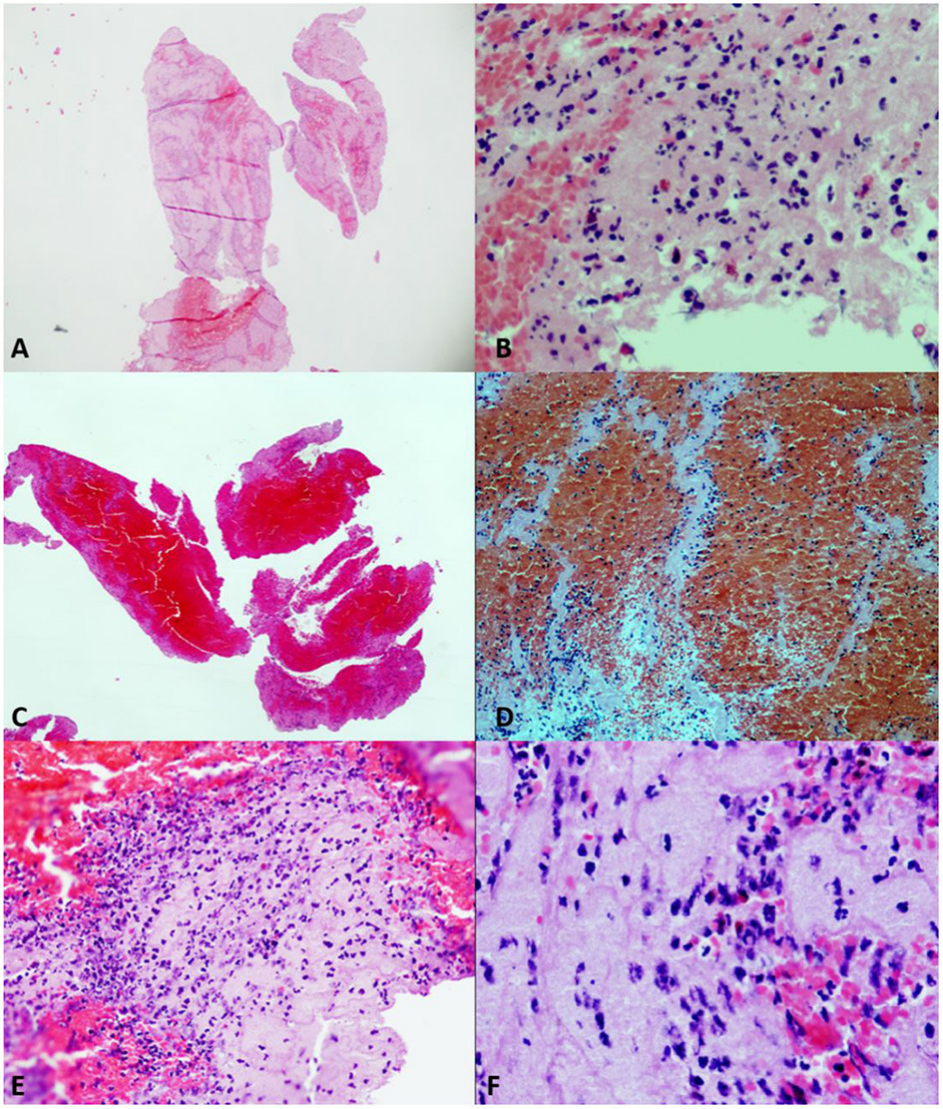
Different samples of clots. Hematoxylin–eosin (HE) stain. **(A)** (HE2×) and **(B)** (HE20×). Platelet fibrin-predominant clot (FPC) with moderate number of complete neutrophils. **(C)** (HE2×) and **(D)** (HE20×). Red cell-predominant clot (RPC), with poorly formed Zahn lines. **(E)** (HE20×) and **(F)** (HE40×). Mixed clot (MC) with plenty of neutrophils, most of them of degenerated appearance.

The histological distribution of the clots was as follows: FPC: 100 (53%), MC: 46 (24%), and RPC: 43 (23%). Figure 3 shows examples of the histological types analyzed.

### Primary Outcome

#### Bivariate Analysis of FPC

FPC patients were more frequently under oral anticoagulation (*p* = 0.042) and had higher levels of CRP (*p* = 0.02). On the contrary, they received less IV thrombolysis (*p* = 0.047) compared with the rest of the cohort and had lower frequency of HMCA in baseline CT (*p* = 0.039). FPC patients had higher rates of mortality (*p* = 0.012) (Table 1).

**Table 1:** Distribution of clinical, laboratory, and radiological variables in patients with fibrin-predominant clot (FPC) vs. the rest.

	**FPC, *n* = 100 (%)**	**Rest, *n* = 89 (%)**	***p***
Age, mean (SD)	69.5 (13)	69.6 (11.4)	0.97
Sex, male	50 (50)	50 (56.2)	0.47
Arterial hypertension	75 (75)	61 (68.5)	0.34
Diabetes mellitus	30 (30)	23 (25.8)	0.63
Dyslipidemia	41 (41)	37 (41.6)	1
Ischemic heart disease	11 (11)	10 (11.2)	1
Smoking	11 (11)	16 (18)	0.17
Oral anticoagulation	32 (32)	17 (19.1)	**0.042**
Atherothrombotic	14 (14)	13 (14.6)	1
Cardioembolic	63 (63)	48 (53.9)	0.24
Baseline NIHSS, median (IQ)	17 (13–23)	18 (10–24)	0.7
Glucose mg/dL, mean (SD)	138.4	138.9	0.95
WBC/mm^3^, mean (SD)	9.228 (2,961)	9.777 (3,158)	0.22
Neutrophils /mm^3^, mean (SD)	6,454 (2,908)	7,285 (3,436)	0.073
Lymphocytes /mm^3^, mean (SD)	1,747 (1,007)	1,784 (895)	0.79
Ratio Neutrophils / Lymphocytes, mean (SD)	5.6 (5.6)	5.5 (4.4)	0.94
Monocytes /mm^3^, mean (SD)	479 (194)	463 (216)	0.61
Platelets /mm^3^, mean (SD)	223,010 (67,274)	219,750 (93,025)	0.78
Fibrinogen mg/dL, mean (SD)	322.9	338.8	0.58
CRP ^*^ mg/dL, mean (SD)	51.5 (61.7)	32.3 (32.3)	**0.02**
Tandem occlusion	16 (16)	19 (21.3)	0.35
Distal occlusion (M2+M3)	24 (24)	14 (15.7)	0.15
ASPECTS, median (IQ)	9 (8–10)	9 (8–10)	0.7
HMCA	55 (55)	61 (68.5)	**0.039**
HU pathological side, mean (SD)	52.8 (9.4)7	54.4 (9.3)	0.35
HU ratio, mean (SD)	1.44 (0.3)7	1.43 (0.4)	0.94
Mismatch, ^**^mean (SD)	80.8 (20)7	81.6 (18.7)	0.8
Alteplase IV	32 (32)	41 (46.1)	**0.047**
PH-2	3 (3)	1 (1.1)	0.36
Onset-CT, mean min (SD)	375 (470.1)	377.4 (480.4)	0.97
Onset-groin, mean min (SD)	496.1 (602.1)	407.1 (470.7)	0.26
Procedure time, mean min (SD)	36.1 (25.2)	37.8 (25.3)	0.63
Number of passes, mean (SD)	1.8 (1.2)	1.7 (1.2)	0.6
Single-pass recanalization	43 (43)	32 (36)	0.32
TICI 2B	13 (61.9)	87 (51.8)	0.49
Stent retriever	85 (85)	72 (80.9)	0.71
Aspiration	13 (13)	14 (15.7)	0.6
mRS ≤ 2 at 3 months	68 (68)	60 (60)	0.93
Mortality	11 (11)	2 (2.2)	**0.012**

* *Calculated over a total of 147 (77.8%) patients with CRP measured after the endovascular procedure*.

** *Calculated over a total of 140 (74.1%) patients with a measurable perfusion study*.

*ASPECTS, Alberta Stroke Protocol Early CT Score; CRP, C-reactive protein; CT, computed tomography; DLP, dyslipidemia; DM, diabetes mellitus; FPC, fibrin-predominant clot; HBP, high blood pressure; HMCA, hyperdensity of the middle cerebral artery; HU, Hounsfield units; IQ, interquartile range; IV, intravenous; M2, 2nd segment of the middle cerebral artery; M3, 3rd segment of the middle cerebral artery; mRS, modified Rankin score; NIHSS, National Institute of Health Stroke Scale; OAC, oral anticoagulation; PH-2, type 2 parenchymal hemorrhagic transformation; SD, standard deviation; TICI, thrombolysis in cerebral infarction; WBC, white blood cell. The bold values indicated to highlight the results*.

#### Bivariate Analysis of Patients Treated With IV Thrombolysis

Seventy-three (39%) of the patients received IV thrombolysis bridging EVT. Thirty-two (44%) had FPC. In a bivariate analysis (Table 2), patients with FPC treated with IV thrombolysis obtained higher rates of complete recanalization compared to the rest of the patients (*p* = 0.005).

**Table 2:** Bivariate analysis: patients treated with intravenous (IV) alteplase previous endovascular treatment (EVT).

	**FPC, *n* = 32 (%)**	**Rest, *n* = 41 (%)**	***p***
Tandem occlusion	4 (13)	6 (15)	1
Single-pass recanalization	10 (31)	17 (42)	0.47
TICI 3	28 (88)	23 (56)	**0.005**
TICI 2B	1 (3)	4 (10)	0.38
Stent retriever as device of choice	30 (94)	34 (83)	0.28
Aspiration as device of choice	2 (6)	6 (15)	0.45
Procedure time, mean min (SD)	30 (25)	35.2 (22)	0.35
Number of passes, mean (SD)	1.4 (1)	1.8 (1)	0.14

*FPC, fibrin-predominant clot; SD, standard deviation; TICI, thrombolysis in cerebral infarction. The bold values indicated to highlight the results*.

#### First-Line Devices in Patients With Fibrin-Predominant Clot (FPC)

For patients with FPC, SR were used as the first-line option in 85 cases (85%), whereas direct aspiration and combined use of both devices were used in 13 (13%) and 2 (2%), respectively. First-pass recanalization was achieved in 50 patients (58.8%) treated with SR as first-line device, six cases with direct aspiration (46.2%), and one case with the combination (50%). No significant differences were found regarding first-pass recanalization. However, SR as the device of choice were more successful in achieving full recanalization (68.2%, *p* = 0.039) compared to direct aspiration (46.2%) or a combination of both (0%) (Table 3).

**Table 3:** Distribution of first-line devices in patients with fibrin-predominant clot (FPC).

	**First-pass effect**	***p***	**TICI2B**	***P***	**TICI 2C**	***p***	**TICI 3**	***p***
**Device of choice, *N* (%)**		0.17		0.24		0.24		**0.009**
Trevo	30 (61.2)		4 (8.2)		45 (91.8)		37 (75)	
Solitaire	14 (63.6)		4 (18.2)		18 (81.8)		13 (59)	
Penumbra	6 (46.2)		3 (23.1)		10 (76.9)		6 (42.6)	
Embotrap	6 (66.6)		0 (0)		9 (100)		7 (77.8)	
Catch mini	0 (0)		0 (0)		2 (100)		0 (0)	
**Stent + aspiration *N* (%)**	1 (50)		1 (50)		1 (50)		0 (0)	
Revive	0 (0)		1 (50)		1 (50)		0 (0)	
Tigertriever	0 (0)		0 (0)		0 (0)		1 (100)	
**Stent retriever, *N* (%)**	50 (58.8)	0.68	9 (10.6)	0.22	76 (89.4)	0.22	58 (68.2)	**0.039**
**Aspiration, *N* (%)**	6 (46.2)	0.55	3 (23.1)	0.37	10 (76.9)	0.37	6 (42.6)	0.22

*TICI, thrombolysis in cerebral infarction. The bold values indicated to highlight the results*.

#### Multivariate Analysis: Predictors of FPC

In a multivariate analysis the absence of HMCA (OR = 0.420; 95% CI 0.197–0.898; *p* = 0.025) and higher levels of CRP after EVT (OR = 1.01; 95% CI 1.003–1.019; *p* = 0.008) were predictors of FPC (Table 4).

**Table 4:** Multivariate analysis: predictors of the fibrin-predominant clot (FPC) in all cohorts.

	**Exp(B)**	**OR (IC 95%)**	***P*-value**
Onset-groin time, mean min (SD)	1	0.999–1.001	0.68
**HMCA**	**0.420**	**0.197–0.898**	**0.025**
OAC	2.114	0.873–5.121	0.097
Alteplase IV	0.534	0.245–1.163	0.11
**CRP**	**1.011**	**1.003–1.019**	**0.008**
Mortality	3.552	0.634–19.902	0.149
Neutrophils	1	1	0.042

*CRP, C-reactive protein; Exp(B), exposure factor; FPC, fibrin-predominant clot; HMCA, hyperdensity of the middle cerebral artery; IV, intravenous; OAC, oral anticoagulation; SD, standard deviation; TICI, thrombolysis in cerebral infarction. The bold values indicated to highlight the results*.

The ROC curve did not disclose any significant association between levels of CRP after the EVT and the histological composition of the clots (area under the curve 0.6, CI 0.5–0.7, *p* = 0.18).

### Secondary Outcome

#### Bivariate Analysis of Functional Outcome and Mortality at 3 Months

In bivariate analysis, higher baseline NIHSS and lower ASPECTS on admission were related to poor functional outcome. Higher levels of baseline glucose and CRP, tandem occlusion, TICI 2B recanalization, and PH-2 were also associated with mRS > 2, as shown in Table 5.

**Table 5:** Bivariate analysis. Functional independence at 3 months and mortality.

	**mRS ≤2, *n* = 128**		***P***	**Mortality, *n* = 13**		***p***
	**Yes**	**No**		**Yes**	**No**	
	***n* = 128 (%)**	***n* = 61 (%)**		***n* = 13 (%)**	***n* = 176 (%)**	
Age, mean (SD)	69.2 (13)	70.3 (11)	0.58	74.2 (7.9)	69.2 (12)	0.15
Sex (male)	63 (49)	37 (61)	0.16	8 (62)	92 (52)	0.58
Arterial Hypertension	87 (64)	49 (80)	0.08	11 (85)	125 (85)	0.36
Diabetes mellitus	21 (34)	32 (25)	0.22	3 (23)	50 (28)	1
Dyslipidemia	31 (51)	47 (37)	0.08	8 (62)	70 (40)	0.15
Ischemic heart disease	10 (16)	11 (9)	0.14	1 (8)	20 (11)	1
Smoking	19 (15)	8 (13)	0.93	0 (0)	27 (15)	0.11
Oral anticoagulation	36 (28)	13 (21)	0.38	5 (37)	44 (25)	0.33
Atherothrombotic	15 (12)	12 (20)	0.18	1 (8)	26 (15)	0.67
Cardioembolic	79 (62)	32 (53)	0.27	10 (77)	101 (58)	0.24
Baseline NIHSS, median (IQ)	16 (11–21)	20 (15–25)	**<0.0001**	19 (17–23)	17 (12–23)	0.18
Glucose mg/dL, mean (SD)	130.9 (41)	155.2 (78)	**0.026**	136.8 (36)	138.8 (58)	0.9
WBC/mm^3^, mean (SD)	9,166 (2,712)	10175.2 (3,646)	0.059	8693.1 (3315.5)	9547.4 (3050.3)	0.33
Neutrophils/mm^3^, mean (SD)	6,321 (2,706)	7,946 (3,806)	**0.004**	6,831 (3,152)	7,045 (3,759)	0.82
Lymphocytes/mm^3^, mean (SD)	1,873 (1,017)	1,537 (763)	**0.012**	1,820 (960)	1,016 (392)	**<0.0001**
Ratio of neutrophils/lymphocytes, mean (SD)	4.8 (4.1)	7.1 (6.5)	**0.014**	5.3 (4.7)	8.9 (8.6)	0.16
Monocytes/mm^3^, mean (SD)	460 (188)	497 (235)	0.25	471 (203)	483 (231)	0.84
Platelets/mm^3^, mean (SD)	216,271 (65,989)	232,393 (103,787)	0.2	222,498 (81,815)	207,615 (54,768)	0.52
Fibrinogen mg/dL, mean (SD)	338.5 (71)	330.5 (75)	0.49	317.4 (68)	337.4 (73)	0.36
CRP * mg/dL, mean (SD)	33.7 (36)	60.3 (71)	**0.016**	38 (35)	42.8 (53)	0.7
Tandem occlusion	16 (46)	19 (54)	**0.004**	2 (15)	33 (19)	1
Distal occlusion (M2+M3)	31 (24)	7 (12)	0.05	1 (8)	37 (21)	0.47
ASPECTS, median (IQ)	9 (8–10)	8 (7–9)	**<0.0001**	8 (7–9)	9 (8–10)	0.21
HMCA	84 (66)	32 (53)	0.11	5 (39)	111 (63)	0.14
HU pathological side, mean (SD)	53.6 (9.5)	53.5 (9)	0.96	58.6 (9.7)	53.2 (9.3)	0.11
HU ratio, mean (SD)	1.4 (0.3)	1.4 (0.4)	0.68	1.6 (0.6)	1.4 (0.3)	0.49
Mismatch**, mean (SD)	83.4 (19)	76.9 (20)	0.05	78 (18.4)	81.4 (19.4)	0.63
Intravenous thrombolysis	51 (40)	22 (36)	0.64	5 (39)	68 (39)	1
PH-2	0	4 (7)	**0.01**	2 (15)	2 (1)	**0.024**
Onset-CT, mean min (SD)	321.3 (430.8)	429.4 (550)	0.14	292.5 (284)	360.9 (485.1)	0.62
Onset-groin, mean min (SD)	394.8 (444.7)	579 (698.1)	0.06	384.2 (323.8)	459.4 (557.7)	0.63
Procedure time, mean min (SD)	35.6 (25)	39.8 (27)	0.28	41.9 (35)	35.6 (24)	0.6
Number of passes, mean (SD)	1.7 (1.3)	1.8 (1.1)	0.71	1.8 (1)	1.8 (1.2)	0.97
First pass effect	28 (45.9)	47 (36.7)	0.27	6 (46.2)	69 (39.2)	0.77
TICI 2B	6 (4.7)	15 (24.6)	**<0.0001**	5 (38.5)	16 (9.1)	**0.007**
Stent retriever	107 (84)	48 (79)	0.42	12 (92)	143 (81)	0.47
Aspiration	17 (13)	10 (16)	0.66	0 (0)	27 (15)	0.22
FPC	68 (53)	32 (53)	1	11 (85)	89 (51)	**0.021**

*ASPECTS, Alberta Stroke Protocol Early CT Score; CRP, C-reactive protein; CT, computed tomography; DLP, dyslipidemia; DM, diabetes mellitus; FPC, fibrin-predominant clot; HBP, high blood pressure; HMCA, hyperdensity of the middle cerebral artery; HU, Hounsfield units; IQ, interquartile range; IV, intravenous; M2, 2nd segment of the middle cerebral artery; M3, 3rd segment of the middle cerebral artery; mRS, modified Rankin score; NIHSS, National Institute of Health Stroke Scale; OAC, oral anticoagulation; PH-2, type 2 parenchymal hemorrhagic transformation; SD, standard deviation; TICI, thrombolysis in cerebral infarction; WBC, white blood cell. The bold values indicated to highlight the results*.

Patients with TICI 2B recanalization, PH-2, and FPC (*p* = 0.021) had higher rates of mortality at 3 months (Table 5).

The absolute number of neutrophils, lymphocytes, and their ratio (neutrophils/lymphocytes) were significantly associated with prognosis at 3 months, while a significant relationship was detected between the absolute number of lymphocytes and mortality (Table 5). None of the cell series analyzed were significantly related to the predominant histological composition (Table 1).

## Discussion

Our series provided a substantial amount of histopathological data about heterogeneous clot samples, as well as clinical, laboratory, neuroimaging, and angiographic information, which allowed us to perform several comparisons between histological subtypes of the thrombus with clinical, laboratory, and radiological variables and recanalization strategies.

Our main finding was the association between patients with FPC and absence of HMCA in their baseline CT scan. In line with our results, a systematic review grouping neuroimaging studies by histological characteristics demonstrated that patients with signs of HCMA on the baseline CT were more likely to have RPC (OR 9.0, 95% CI 2.6–31.2, *p* < 0.01) (17), but not FPC.

We also detected a significant association between levels of CRP after EVT and the presence of FPC. Other studies looking at coronary artery disease (19) and deep venous thrombosis (20) have shown that CRP-level increases were associated with FPC formation. A study performed in patients with acute coronary syndrome (21) has shown a strong association between CRP levels and resistance to FPC lysis, so acute-phase reactants could act as platelet aggregation and clot hardness markers. With regard to the existing association with CRP levels, it must be clarified that in our series these analyses were obtained immediately after the procedures, so it cannot be considered as a baseline biomarker of the patients' condition. No significant association was found between leukocyte count and FPC. Consistent with previous studies (22), higher neutrophil counts and relative neutrophilia on admission were associated with bad functional outcome, while relative lymphocytosis corresponded with higher mortality.

To evaluate the effect of IV thrombolysis, we performed a subanalysis in patients with FPC and we found that these patients had higher complete recanalization rates. These data suggest a synergistic effect of the IV thrombolysis bridging EVT in patients with FPC and reinforce the indication of current guidelines giving IV thrombolysis if no contraindication exists (23).

In our study, FPC patients had higher mortality rates compared to the rest of the cohort; these patients were more frequently under oral anticoagulation and received less intravenous thrombolysis as expected. Regarding thrombectomy devices, patients with FPC achieved full recanalization (TICI 3) with higher probability when SR were selected as first-line treatment.

There are limited studies that have taken into consideration thrombus composition when comparatively analyzing the efficacy of the main recanalization strategies (stent retrieval or aspiration). In particular, the higher coefficient of friction of FPC may increase their resistance to direct suction and may require a higher number of contact points for complete extraction. In line with our results, an experimental study comparing direct aspiration to SR (24), white clots were retrieved by the SR and balloon guide catheter with fewer distal emboli.

The study of the histopathological and mechanical properties of the clot should be part of the strategy for the development of new EVT endovascular devices. Behavior of clots is currently analyzed using experimental models *in vitro* (8, 25), thus trying to obtain better recanalization rates (12). In the clinical setting, the potential to establish predictors of the histological composition of the clots could optimize the selection of reperfusion strategies, as well as extend the use of endovascular devices with a higher efficacy in case of resistant clots.

The fibrin structure present in a clot is a critical element that modifies its mechanical behavior, as it gives it properties of hardness (26), impenetrability (7), and friction, which involve greater technical difficulty (12), lower vascular recanalization rates, and treatment delay (18, 27).

Despite this evidence, uncertainty persists regarding the relationship between clot composition, angiographic outcome, and stroke etiology (17), so the practical value of obtaining the histopathological characteristics of the clot in acute stroke remains unclear.

On the other hand, the analysis of the neuroimaging prior to reperfusion treatment allows the identification of recanalization standards, by means of qualitative signs such as HMCA on the baseline CT (28), or quantitative signs, such as clot permeability obtained by HU measurement (29). However, it has not been established if there are neuroimaging models which allow us to infer the histological composition of the clot, using CT (6, 9), and with magnetic resonance imaging (MRI) (30). As in previous studies (10), we have not been able to establish a direct association between HU determination and the predominant fibrin composition.

This study has some limitations. First, it is a single-center, retrospective series, so the results could be affected by various biases. The determination of a cutoff point of 60% to discriminate the predominance of a histologic subtype by a semiquantitative method makes it difficult to neatly identify subgroups and may alter the analysis. Although we have used several observers to minimize variability in the analysis of images, precision could have been greater if we had recorded the kappa coefficient. CRP was measured only in 78% of the patients and once the EVT was completed, so it cannot be categorized as a baseline biomarker. The ultrasensitive CRP systematic analysis before reperfusion treatment might contribute toward the elucidation of this. We have only included patients with single anterior circulation occlusion and with high recanalization rates, so the sample may have been excessively selected, losing sensitivity in some of the known prognostic variables (as first-pass effect). The higher percentage of anticoagulated patients existing in the FPC group could condition the administration of IV thrombolysis, limiting the scope of the results.

## Conclusions

The absence of HMCA on baseline CT and CRP levels appear to be a reliable marker of FPC, which could allow an individualized treatment approach such as prior administration of intravenous thrombolysis and the use of specific thrombectomy devices. In our series, the choice of a SR as first-line treatment in FPC cases was more effective in achieving complete recanalization than aspiration devices. Histopathological study and identification of treatment-refractory clots improve patient characterization and could guide therapeutic strategies for recanalization.

## Data Availability Statement

The raw data supporting the conclusions of this article will be made available by the authors, without undue reservation.

## Ethics Statement

The studies involving human participants were reviewed and approved by CEIm of University Hospital of Albacete. The patients/participants provided their written informed consent to participate in this study.

## Author Contributions

FH-F conceived the study, drafted the manuscript, and performed the statistical analysis. MER-A drafted the manuscript and performed the statistical analysis. RB-A performed the histopathological analysis and drafted the manuscript. FH-F, MER-A, RB-A, JM, JG-G, OA-M, MP-J, LL-M, GS-H, EJ-M and TS acquired the data. TS critically reviewed the manuscript. All authors contributed to the article and approved the submitted version.

## Conflict of Interest

The authors declare that the research was conducted in the absence of any commercial or financial relationships that could be construed as a potential conflict of interest.

## References

[1] Powers WJ Rabinstein AA Ackerson T Adeoye OM Bambakidis NC Becker K. Guidelines for the early management of patients with acute ischemic stroke: 2019 update to the 2018 guidelines for the early management of acute ischemic stroke: a guideline for healthcare professionals from the American Heart Association/American Stroke Association. Stroke. (2019) 50:344–418. 10.1161/STR.0000000000000211 30626290

[2] Dargazanli C Fahed R Blanc R Gory B Labreuche J Duhamel A. Modified thrombolysis in cerebral infarction 2C/thrombolysis in cerebral infarction 3 reperfusion should be the aim of mechanical thrombectomy: insights from the ASTER trial (contact aspiration versus stent retriever for successful revascularization). Stroke. (2018) 49:1189–96. 10.1161/STROKEAHA.118.020700 29626134

[3] Zaidat OO Castonguay AC Linfante I Gupta R Martin CO Holloway WE. First pass effect: a new measure for stroke thrombectomy devices. Stroke. (2018) 49:660–6. 10.1161/STROKEAHA.117.020315 29459390

[4] Nikoubashman O Dekeyzer S Riabikin A Keulers A Reich A Mpotsaris A. True first-pass effect: first-pass complete reperfusion improves clinical outcome in thrombectomy stroke patients. Stroke. (2019) 50:2140–6. 10.1161/STROKEAHA.119.025148 31216965

[5] García-Tornel Á Requena M Rubiera M Muchada M Pagola J Rodriguez-Luna D. When to stop: detrimental effect of device passes in acute ischemic stroke secondary to large vessel occlusion. Stroke. (2019) 50:1781–8. 10.1161/STROKEAHA.119.025088 31177974

[6] Berndt M Friedrich B Maegerlein C Moench S Hedderich D Lehm M. Thrombus permeability in admission computed tomographic imaging indicates stroke pathogenesis based on thrombus histology. Stroke. (2018) 49:2674–82. 10.1161/STROKEAHA.118.021873 30355200

[7] Benson JC Fitzgerald ST Kadirvel R Johnson C Dai D Karen D. Clot permeability and histopathology: is a clot's perviousness on CT imaging correlated with its histologic composition? J Neurointerv Surg. (2020) 12:38–42. 10.1136/neurintsurg-2019-014979 31239329 PMC7744246

[8] Sanchez S Cortiñas I Villanova H Rios A Galve I Andersson T. ANCD thrombectomy device: *in vitro* evaluation. J Neurointerv Surg. (2020) 12:77–81. 10.1136/neurintsurg-2019-014856 31197024

[9] Hashimoto T Hayakawa M Funatsu N Yamagami H Satow T Takahashi JC. Histopathologic analysis of retrieved thrombi associated with successful reperfusion after acute stroke thrombectomy. Stroke. (2016) 47:3035–7. 10.1161/STROKEAHA.116.015228 27780903

[10] Liebeskind DS Sanossian N Yong WH Starkman S Tsang MP Moya AL. CT and MRI early vessel signs reflect clot composition in acute stroke. Stroke. 2011 May; 42:1237–43. 10.1161/STROKEAHA.110.605576 21393591 PMC3094751

[11] Niesten JM Van Der Schaaf IC Van Dam L Vink A Vos JA Schonewille WJ. Histopathologic composition of cerebral thrombi of acute stroke patients is correlated with stroke subtype and thrombus attenuation. PLoS ONE. (2014) 9:12–4. 10.1371/journal.pone.0088882 24523944 PMC3921255

[12] Duffy S McCarthy R Farrell M Thomas S Brennan P Power S. Per-pass analysis of thrombus composition in patients with acute ischemic stroke undergoing mechanical thrombectomy. Stroke. (2019) 50:1156–63. 10.1161/STROKEAHA.118.023419 31009342

[13] Adams HP Bendixen BH Kappelle LJ Biller J Love BB Gordon DL. Classification of subtype of acute ischemic stroke. Definitions for use in a multicenter clinical trial. TOAST. Trial of Org 10172 in Acute Stroke Treatment. Stroke. (1993) 24:35–41. 10.1161/01.STR.24.1.35 7678184

[14] Von Kummer R Broderick JP Campbell BCV Demchuk A Goyal M Hill MD. The Heidelberg Bleeding Classification: Classification of bleeding events after ischemic stroke and reperfusion therapy. Stroke. (2015) 46:2981–6. 10.1161/STROKEAHA.115.010049 26330447

[15] Leys D Pruvo JP Godefroy O Rondepierre P Leclerc X. Prevalence and significance of hyperdense middle cerebral artery in acute stroke. Stroke. (1992) 23:317–24. 10.1161/01.STR.23.3.317 1542889

[16] Fitzgerald S Wang S Dai D Murphree DH Pandit A Douglas A. Orbit image analysis machine learning software can be used for the histological quantification of acute ischemic stroke blood clots. PLoS ONE. (2019) 14:1–14. 10.1371/journal.pone.0225841 31805096 PMC6894878

[17] Brinjikji W Duffy S Burrows A Hacke W Liebeskind D Majoie CBLM. Correlation of imaging and histopathology of thrombi in acute ischemic stroke with etiology and outcome: a systematic review. J Neurointerv Surg. (2017) 9:529–34. 10.1136/neurintsurg-2016-012391 27166383 PMC6697418

[18] Boeckh-Behrens T Schubert M Förschler A Prothmann S Kreiser K Zimmer C. The impact of histological clot composition in embolic stroke. Clin Neuroradiol. (2016) 26:189–97. 10.1007/s00062-014-0347-x 25261075

[19] Lv HC Wu HY Yin JS Ge JB. Thrombin induced platelet-fibrin clot strength in relation to platelet volume indices and inflammatory markers in patients with coronary artery disease. Oncotarget. (2017) 8:64217–23. 10.18632/oncotarget.19450 28969064 PMC5609996

[20] Wójcik M Zareba L Undas A. Prothrombotic fibrin clot properties are associated with post-discharge venous thromboembolism in acutely ill medical patients. Thromb Res. (2019) 182:141–9. 10.1016/j.thromres.2019.08.010 31479942

[21] Sumaya W Wallentin L James SK Siegbahn A Gabrysch K Bertilsson M. Fibrin clot properties independently predict adverse clinical outcome following acute coronary syndrome: a PLATO substudy. Eur Heart J. (2018) 39:1078–85. 10.1093/eurheartj/ehy013 29390064 PMC6019045

[22] Semerano A Laredo C Zhao Y Rudilosso S Renú A Llull L. Leukocytes, collateral circulation, and reperfusion in ischemic stroke patients treated with mechanical thrombectomy. Stroke. (2019) 50:3456–64. 10.1161/STROKEAHA.119.026743 31619153

[23] Turc G Bhogal P Fischer U Khatri P Lobotesis K Mazighi M. European Stroke Organisation (ESO) – European Society for Minimally Invasive Neurological Therapy (ESMINT) guidelines on mechanical thrombectomy in acute ischaemic Stroke Endorsed by Stroke Alliance for Europe (SAFE). Eur Stroke J. (2019) 4:6–12. 10.1177/2396987319832140 31165090 PMC6533858

[24] Madjidyar J Pineda Vidal L Larsen N Jansen O. Influence of thrombus composition on thrombectomy: ADAPT vs. balloon guide catheter and stent retriever in a flow model. Rofo. (2020) 192:257–263. 10.1055/a-0998-4246 31514211

[25] Fennell VS Setlur Nagesh SV Meess KM Gutierrez L James RH Springer ME. What to do about fibrin rich “tough clots”? Comparing the Solitaire stent retriever with a novel geometric clot extractor in an *in vitro* stroke model. J Neurointerv Surg. (2018) 10:907–10. 10.1136/neurintsurg-2017-013507 29352061

[26] Johnson S Chueh J Gounis MJ McCarthy R McGarry JP McHugh PE. Mechanical behavior of *in vitro* blood clots and the implications for acute ischemic stroke treatment. J Neurointerv Surg. (2020) 12:853–7. 10.1136/neurintsurg-2019-015489 31780453

[27] Yuki I Kan I Vinters HV Kim RH Golshan A Vinuela FA. The impact of thromboemboli histology on the performance of a mechanical thrombectomy device. Am J Neuroradiol. (2012) 33:643–8. 10.3174/ajnr.A2842 22207297 PMC8050469

[28] Li Q Davis S Mitchell P Dowling R Yan B. Proximal hyperdense middle cerebral artery sign predicts poor response to thrombolysis. PLoS ONE. (2014) 9:e96123. 10.1371/journal.pone.0096123 24804962 PMC4013049

[29] Santos EMM Dankbaar JW Treurniet KM Horsch AD Roos YB Kappelle LJ. Permeable thrombi are associated with higher intravenous recombinant tissue-type plasminogen activator treatment success in patients with acute ischemic stroke. Stroke. (2016) 47:2058–65. 10.1161/STROKEAHA.116.013306 27338928

[30] Kim SK Yoon W Kim TS Kim HS Heo TW Park MS. Histologic analysis of retrieved clots in acute ischemic stroke: correlation with stroke etiology and gradient-echo MRI. AJNR Am J Neuroradiol. (2015) 36:1756–62. 10.3174/ajnr.A4402 26159515 PMC7968760

